# Genome-wide identification and functional analysis of U-box E3 ubiquitin ligases gene family related to drought stress response in Chinese white pear (*Pyrus bretschneideri*)

**DOI:** 10.1186/s12870-021-03024-3

**Published:** 2021-05-26

**Authors:** Chunmeng Wang, Bobo Song, Yuqin Dai, Shaoling Zhang, Xiaosan Huang

**Affiliations:** Center of Pear Engineering Technology Research, State Key Laboratory of Crop Genetics and Germplasm Enhancement, Nanjing Agricultural University, Nanjing, 210095 China

**Keywords:** Ubiquitin ligases, *PUB* gene family, *Pyrus*, *PbrPUB18*, Abiotic stresses

## Abstract

**Background:**

The plant U-box (PUB) proteins are a family of ubiquitin ligases (E3) enzymes that involved in diverse biological processes, as well as in responses to plant stress response. However, the characteristics and functional divergence of the *PUB* gene family have not yet been previously studied in the Chinese white pear (*Pyrus bretschneideri*).

**Results:**

In the present study, we identified 62 *PbrPUBs* in Chinese white pear genome. Based on the phylogenetic relationship, 62 *PUB* genes were clustered into five groups. The results of conserved motif and gene structure analysis supported the classification phylogenetic tree. The *PbrPUB* genes were unevenly distribution on 17 pear chromosomes, chromosome 15 housed most member of *PUB* family, with eight *PUB* genes. *Cis*-acting element analysis indicated that *PUB* genes might participate in diverse biological processes, especially in the response to abiotic stresses. Based on RNA-data from ‘Dangshansuli’ at seven tissues, we found that *PUB* genes exhibited diverse of expression level in seven tissues, and qRT-PCR experiment further supported the reliable of RNA-Seq data. To identify candidate genes associated with resistance, we conducted qRT-PCR experiment the expression level of pear seed plant under four abiotic stresses, including: ABA, dehydration, salt and cold treatment. One candidate *PUB* gene associated with dehydration stress was selected to conduct further functional experiment. Subcellular localization revealed PbrPUB18 protein was located on cell nucleus. Furthermore, heterologous over-expression of *PbrPUB18* in *Arabidopsis* indicated that the over-expression of *PbrPUB18* could enhance resistance in drought treatment. In conclusions, we systematically identified the *PUB* genes in pear, and provided useful knowledge for functional identification of *PUB* genes in pear.

**Supplementary Information:**

The online version contains supplementary material available at 10.1186/s12870-021-03024-3.

## Background

Plants are frequently exposed to various abiotic stresses such as drought, salt and low temperature during their life cycles. Several stresses often lead to oxidative damage and have adverse impacts on plant growth and development. To adapt to unfavorable environmental conditions, plants have evolved complex and efficient mechanisms [1]. Previous studies have identified four signal transduction pathways in response to abiotic stress, including transcriptional regulation, post-transcriptional modifications, epigenetic regulation, and post-translational modifications [2]. And ubiquitination is one of the most significant post-translational modifications. The ubiquitin/26S proteasome system (UPS) pathway is a pervasive and effective route for protein removal in eukaryotes [3, 4]. UPS include ubiquitin (Ub), ubiquitin-activating enzyme (E1), ubiquitin- conjugating enzyme (E2), ubiquitin ligase (E3), and the 26S proteasome. The central component of UPS is the highly conserved, 76 amino acid protein ubiquitin. Ubiquitin is bound to specific proteins and functions in the degradation of target proteins in an E1–E2–E3 multienzyme cascade manner [5–8]. In the pathway, E3 enzymes are clearly the key factors that define substrate specificity. According to their reaction mechanism and subunit compositions, four main types were classified: U­box, HECT (Homology to E6-Associated Carboxy-Terminus), RING (Really Interesting New Gene) and Cullin–RING ligases (CRLs) [4]. U-box ubiquitin ligases are characterized by a conserved U-box motif of about 70 amino acids. And U-box ubiquitin ligases were firstly discovered among E3 ubiquitin ligases, and was first clarified from ubiquitin fusion degradation protein-2 (UFD2) in yeast [9].

In comparison with the 2 and 21 U-box (PUB) genes identified in *Saccharomyces cerevisiae* and *Homo sapiens* genomes, respectively, more U-box genes were widely distributed in plants. In *Arabidopsis thaliana*, about 61 plant U-box genes were predicted [9, 10], while 77 were found in *Oryza sativa* [11], 62 in *Solanum lycopersicum* [12], 93 in *Gossypium raimondii* [13], 91 in *Musa acuminate* [14], 61 in *Medicago truncatula* [15] 101 in *Brassica rapa* [16] and 125 in soybean [17]. In apple[18], 69 *PUB* genes were identified, and were divided into seven subgroups based on phylogenetic tree of PUB proteins from apple and *Arabidopsis thaliana*. Many previous studies have shown that PUB proteins are involved in biological processes such as plant hormone signaling regulations [6], self-incompatible or pseudo-self-compatibility regulations [19] as well as in biotic stress [20–22] and abiotic stress [5, 23, 24].

Drought is one of most threatening factors influencing the yield of agronomic crops in the world. Thus, it is certainly need to comprehend the molecular mechanisms of plant response to drought stress and develop drought resistant crops. The signaling pathways induced by drought stress include signal perception, signal transduction and response, as well as the activation of metabolic and physiological reactions [25, 26]. E3 ubiquitin ligases may play a role by inhibiting the drought stress signaling pathway under favorable growth conditions. They may eliminate negative regulators of the stress signaling pathway in response to stimulation or reduce, and eliminate the signaling pathway in time after stress conditions disappear to maintain plants further growth. It is also possible that E3 ubiquitin ligases may act as a positive feedback factor to enhance stress signaling [27]. In *Arabidopsis*, *PUB11* negatively modulated drought responses by ubiquitin mediated degradation of the receptor like protein kinases LRRR1 (LEUCINE‐RICH REPEAT PROTEIN1) and KIN7 (KINASE7) [28]. A previous study shown that *PUB12* and *PUB13* affect ABA-mediated drought tolerance through targeting ABI1 (ABA-INSENSITIVE 1) [29].

In generally, the activity of transcription factors were regulated by upstream components. After modifications of sumoylation and ubiquitination, they form a complex regulatory network to effect the expression level of genes involved in stress, and then regulate several metabolic and physiological processes [30, 31]. *PUB25* and *PUB26*, two U-box type E3 ubiquitin ligases, trigger cold signaling negative regulator *MYB15* to promote plant freezing tolerance [32]. In a number of previous studies, U-box genes acted as regulators in diverse abiotic stress responses including drought, low temperature and salinity conditions. In *Arabidopsis thaliana*, *AtPUB18* and *AtPUB19* are negative regulators of ABA signaling by inducing ABA hypersensitivity, and *AtPUB22/AtPUB23* are negative regulators in drought stress responses in an ABA-independent pathway [33, 34]. *AtPUB44* ubiquitinates the AAO3 (abscisic aldehyde oxidase 3) via 26 proteasome and affects the ABA biosynthesis [35]. Furthermore, *AtPUB46* and *AtPUB48* were found to be more sensitive to drought [36]. In rice, *OsPUB15* has been implicated in positive regulating plant tolerance to salinity and drought stresses [37]. In apple, *MdPUB29* may positively regulate salt tolerance [38].

The plant PUB family has been widely studied for abiotic stresses, mainly in model plants such as *Arabidopsis*, rice and tomato, and less on woody plants such as pear. Pear belongs to the *Pyrus* genus in the Rosaceae family, and is one of the most important fruit crops and widely distributed fruits in the world. However, due to the effects of abiotic stresses, yield of pear frequently came down. And these abiotic stresses affect growth and development of pear trees, furthermore limit pear crop productivity [39]. Therefore, it is significant to identify genetic determinants associated with drought, cold and salinity stresses tolerance in pear for agricultural development. Based on the Chinese white pear (*Pyrus bretschneideri*) genome [40], we conducted systematic characterization of *PUB* genes, and further verified the function of *PbrPUB18* associated with drought stress. These results provided new insights for function verification of *PUB* gene in future.

## Results

### Identification of *PbrPUB* gene family members

In our study, we used a strictly pipeline to identify *PUB* genes in pear genome (See Methods). As a result, a total of 91 candidate *PUB* genes were identified in pear genome. SMART tools were performed to verify the accuracy of 91 candidate *PUB* genes, and we deleted 29 *PUB* genes lacked of U-box domain. At last, 62 *PUB* genes with complete U-box domain were obtained for further analysis. The number of *PUB* genes in pear are similar to the number of *PUB* genes in apple (69) [18]. Based on the location order of *PUB* genes, we named 62 *PbrPUB* genes (Table 1). The molecular weight (MW) for the *PbrPUB* gene family range from 39.33 kDa to 151.30 kDa (Kilodalton) and the isoelectric points (pI) range from 4.99 to 8.83, with an average of 6.78. Subcellular localization of *PbrPUBs* were also predicted by Cell-PLoc 2.0, and we found that most PUB proteins were located on cell nucleus, except six located in cytoplasm and three located in cell membrane (Table 1).

**Table 1:** The members of *PbrPUB* gene family in Chinese white pear (*Pyrus bretschneideri*) genome

Gene ID	Name	Chromosome	Localization		Gene DNA (bp)	CDS (bp)	Protein Length (aa)	Molecular Weight(kDa)	Theoretical pI	Putative Subcellular Localization
			Start	End						
Pbr015706.1	*PbrPUB1*	Chr1	256,929	261,486	4558	2337	779	86.85	7.14	Nucleus
Pbr018393.1	*PbrPUB2*	Chr1	4,223,236	4,229,195	5960	3018	1006	111.54	5.31	Cytoplasm. Nucleus
Pbr010707.1	*PbrPUB3*	Chr1	4,989,120	4,992,958	3839	1881	627	68.67	5.52	Nucleus
Pbr009550.1	*PbrPUB4*	Chr1	7,179,899	7,181,971	2073	2073	691	75.68	7.42	Nucleus
Pbr001047.1	*PbrPUB5*	Chr2	11,921,509	11,926,006	4498	1794	598	65.12	7.87	Cytoplasm. Nucleus
Pbr025219.1	*PbrPUB6*	Chr2	12,726,277	12,735,326	9050	2496	832	90.62	5.48	Nucleus
Pbr032056.1	*PbrPUB7*	Chr2	15,355,717	15,361,035	5319	2268	756	84.21	6.51	Nucleus
Pbr041491.1	*PbrPUB8*	Chr2	18,417,877	18,422,209	4333	2271	757	84.62	6.89	Nucleus
Pbr022832.1	*PbrPUB9*	Chr3	1,830,500	1,832,044	1545	1341	447	49.96	6.65	Nucleus
Pbr013414.1	*PbrPUB10*	Chr3	20,415,253	20,417,829	2577	1890	630	68.32	7.89	Nucleus
Pbr028559.1	*PbrPUB11*	Chr4	334,757	337,647	2891	1416	472	51.45	6.78	Nucleus
Pbr010503.2	*PbrPUB12*	Chr5	2,805,518	2,807,272	1755	1248	416	44.82	7.71	Nucleus
Pbr031387.1	*PbrPUB13*	Chr5	19,974,875	19,977,231	2357	1371	457	50.70	8.17	Cytoplasm. Nucleus
Pbr031385.1	*PbrPUB14*	Chr5	19,988,832	19,991,731	2900	1047	349	39.33	5.33	Nucleus
Pbr026702.1	*PbrPUB15*	Chr5	20,027,000	20,030,429	3430	2355	785	85.43	7.31	Nucleus
Pbr000535.1	*PbrPUB16*	Chr5	24,705,275	24,708,248	2974	2154	718	78.13	6.45	Nucleus
Pbr000230.1	*PbrPUB17*	Chr5	26,978,015	26,979,592	1578	1146	382	41.72	7.52	Nucleus
Pbr028339.1	*PbrPUB18*	Chr6	2,556,224	2,557,558	1335	1335	445	49.74	6.2	Nucleus
Pbr015077.1	*PbrPUB19*	Chr6	20,252,411	20,257,233	4823	2439	813	88.94	5.35	Nucleus
Pbr031290.1	*PbrPUB20*	Chr7	9,488,534	9,495,611	7078	2679	893	99.85	6.24	Cell membrane. Nucleus
Pbr010929.1	*PbrPUB21*	Chr7	11,579,201	11,581,264	2064	2064	688	75.44	6.63	Nucleus
Pbr040085.1	*PbrPUB22*	Chr7	14,330,366	14,333,661	3296	3006	1002	110.91	5.08	Cytoplasm. Nucleus
Pbr016655.1	*PbrPUB23*	Chr9	2,939,065	2,940,405	1341	1341	447	50.23	7.72	Nucleus
Pbr006392.1	*PbrPUB24*	Chr9	17,767,680	17,773,728	6049	3063	1021	115.77	5.1	Chloroplast. Nucleus
Pbr016092.1	*PbrPUB25*	Chr10	3,280,331	3,283,357	3027	2154	718	78.38	6.45	Nucleus
Pbr014928.1	*PbrPUB26*	Chr10	12,833,713	12,836,171	2459	1371	457	50.67	8.17	Cytoplasm. Nucleus
Pbr024184.1	*PbrPUB27*	Chr10	13,098,222	13,100,641	2420	1371	457	50.61	8.34	Nucleus
Pbr029555.1	*PbrPUB28*	Chr11	14,720,560	14,721,798	1239	1239	413	46.46	8.22	Nucleus
Pbr029558.1	*PbrPUB29*	Chr11	14,803,257	14,804,501	1245	1245	415	46.08	8.55	Nucleus
Pbr023629.1	*PbrPUB30*	Chr12	1,386,012	1,387,978	1967	1410	470	51.42	6.73	Nucleus
Pbr024251.1	*PbrPUB31*	Chr12	13,342,776	13,343,999	1224	1224	408	45.13	6.29	Nucleus
Pbr035713.1	*PbrPUB32*	Chr12	14,483,315	14,484,986	1672	1221	407	45.13	8.55	Nucleus
Pbr001353.1	*PbrPUB33*	Chr12	18,453,593	18,457,109	3517	2385	795	88.75	6.44	Cell membrane. Nucleus
Pbr018623.1	*PbrPUB34*	Chr13	8,122,125	8,126,781	4657	3093	1031	114.59	6.87	Nucleus
Pbr034872.1	*PbrPUB35*	Chr13	14,992,569	14,994,626	2058	2058	686	74.46	8.83	Nucleus
Pbr010331.1	*PbrPUB36*	Chr14	1,982,945	1,984,165	1221	1221	407	45.01	8.41	Nucleus
Pbr010332.1	*PbrPUB37*	Chr14	2,000,839	2,002,376	1538	1212	404	45.81	7.86	Nucleus
Pbr038818.1	*PbrPUB38*	Chr14	16,851,458	16,852,792	1335	1335	445	49.83	6.76	Nucleus
Pbr019772.1	*PbrPUB39*	Chr15	7,027,972	7,030,827	2856	1362	454	48.79	7.32	Nucleus
Pbr015478.1	*PbrPUB40*	Chr15	15,983,582	15,990,594	7013	4104	1368	151.30	5.4	Nucleus
Pbr014329.2	*PbrPUB41*	Chr15	17,789,694	17,794,348	4655	2280	760	85.02	6.42	Nucleus
Pbr004693.1	*PbrPUB42*	Chr15	21,640,514	21,645,193	4680	2403	801	91.56	6.77	Nucleus
Pbr004694.1	*PbrPUB43*	Chr15	21,654,165	21,658,208	4044	2412	804	91.82	8.04	Nucleus
Pbr024318.1	*PbrPUB44*	Chr15	24,513,187	24,517,833	4647	1788	596	65.04	7.88	Cytoplasm. Nucleus
Pbr025038.1	*PbrPUB45*	Chr15	29,959,515	29,960,959	1445	1260	420	46.65	7.48	Nucleus
Pbr020827.1	*PbrPUB46*	Chr15	42,112,935	42,118,262	5328	1986	662	71.75	5.15	Nucleus
Pbr012148.1	*PbrPUB47*	Chr16	3,520,190	3,521,515	1326	1326	442	49.02	7.28	Nucleus
Pbr015195.1	*PbrPUB48*	Chr16	6,260,812	6,266,259	5448	2328	776	85.59	4.99	Nucleus
Pbr001186.1	*PbrPUB49*	Chr16	19,513,720	19,518,315	4596	2442	814	89.22	5.15	Nucleus
Pbr017987.1	*PbrPUB50*	Chr17	19,820,745	19,823,337	2593	1329	443	47.63	6.69	Nucleus
Pbr003423.1	*PbrPUB51*	scaffold1145.0	13,868	15,091	1224	1224	408	45.13	6.29	Nucleus
Pbr003424.1	*PbrPUB52*	scaffold1145.0	87,357	88,580	1224	1224	408	45.13	6.29	Nucleus
Pbr003570.1	*PbrPUB53*	scaffold1158.0	67,952	71,514	3563	3048	1016	112.40	5.26	Nucleus
Pbr007911.1	*PbrPUB54*	scaffold1459.0	15,991	18,530	2540	1590	530	58.02	7.48	Nucleus
Pbr012694.1	*PbrPUB55*	scaffold195.0	243,575	247,284	3710	2337	779	86.98	6.99	Cell membrane. Nucleus
Pbr026868.1	*PbrPUB56*	scaffold435.0.1	119,841	123,521	3681	2511	837	90.94	5.19	Nucleus
Pbr026878.1	*PbrPUB57*	scaffold435.0.1	363,440	367,075	3636	2511	837	90.94	5.19	Nucleus
Pbr028067.1	*PbrPUB58*	scaffold463.0	87,246	88,490	1245	1245	415	46.07	8.55	Nucleus
Pbr028068.1	*PbrPUB59*	scaffold463.0	211,688	212,932	1245	1245	415	46.07	8.55	Nucleus
Pbr028907.1	*PbrPUB60*	scaffold482.0	220,241	221,464	1224	1224	408	45.15	6.29	Nucleus
Pbr036646.1	*PbrPUB61*	scaffold727.0	14,450	19,726	5277	2019	673	72.93	5.39	Nucleus
Pbr036650.1	*PbrPUB62*	scaffold727.0	98,641	103,917	5277	2019	673	72.93	5.39	Nucleus

### Phylogenetic analysis of *PbrPUB* gene family members

**Fig. 1:**
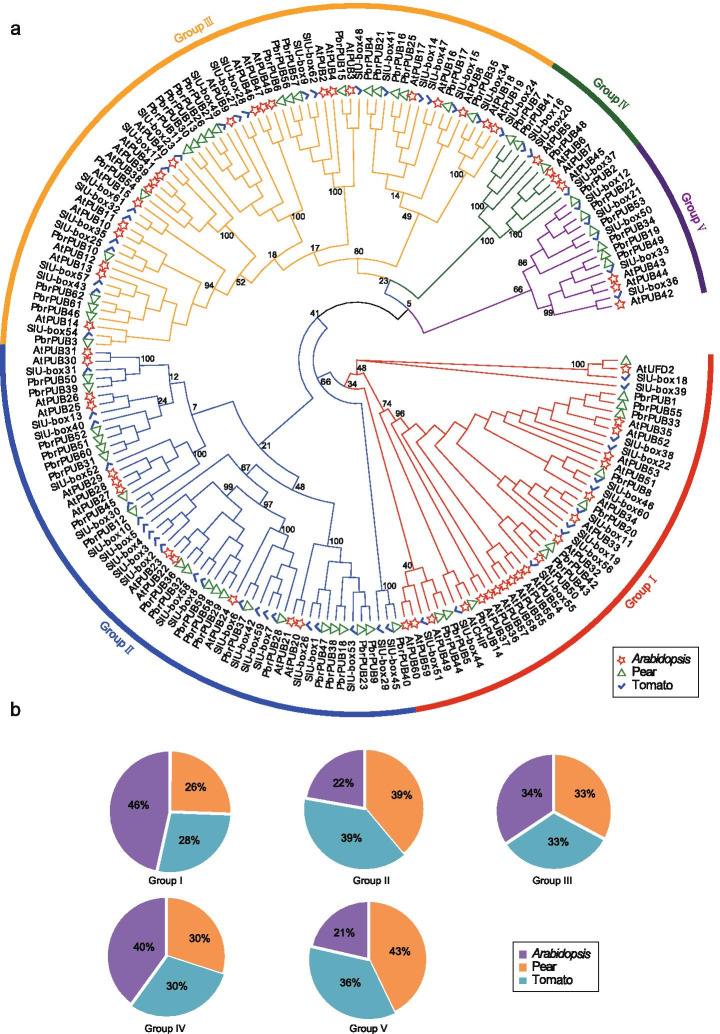
Phylogenetic tree analysis of *PUB* gene family. **a** A Neighbor–Joining (NJ) tree of PUB proteins from three species, including pear, tomato and *Arabidopsis*. The phylogenetic tree was constructed by Mega-X software with 1000 boot strap. The red star, green triangle and blue tick represents the PUB proteins in *Arabidopsis*, pear and tomato, respectively. All of 185 PUB proteins from three species were clustered into five subgroups, named Group I, II, III, IV and V; **b** Five pie plots represented the percentage of *PUB* genes of three species in five groups. The orange part represented the percentage of *PUB* genes in pear, and the blue part represented the percentage of *PUB* genes in tomato, and the purple part presented the percentage of *PUB* genes in *Arabidopsis*

To investigate the evolutionary history of *PUB* genes in pear, we constructed a phylogenetic tree (NJ, neighbor-joining) using the Mega-X tool based on the PUB proteins from pear (62 members), tomato (62 members) and *Arabidopsis* (61 members) (Fig. 1a). The protein sequences of *PUB* genes of tomato and *Arabidopsis* were obtained from previous study [10, 12]. Based the result of phylogenetic tree, 185 members of *PUB* genes from these three species were clustered into five subgroups, including Group I, Group II, Group III, Group IV and Group V. The member number of Group III was the largest in five subgroups, and it harboured 64 *PUB* genes. However, Group IV harboured the least *PUB* genes, with 10 *PUB* genes. In generally, the *PUB* genes of pear and tomato were clustered into one subclade, suggesting that pear and tomato exhibited relatively closer relationship compared to *Arabidopsis*.

It is interesting to note that the number of *PUB* gene family in these three species is similar. This result indicated that the number of *PUB* genes in these three species is conserved. To explore which group of pears had occurred expansion or lost during evolution process, we measure the number of *PUB* genes of each species in each group. In pear, Group I, II, III, IV and V contain 11, 21, 21, 3 and 6 *PbrPUB* gene family members, respectively. In tomato, Group I, II, III, IV and V contain 12, 21, 21, 3, and 5 *SIU-box* genes, respectively. In *Arabidopsis*, Group I, II, III, IV and V contain 20, 12, 22, 4 and 3 *AtPUB* genes, respectively (Fig. 1b). The member number of each group in pear and tomato is almost equal, suggesting that pear had not undergone expansion or lost compared to tomato. However, compared to pear and tomato, the group I of *Arabidopsis* had undergone rapid expansion, while the group II of *Arabidopsis* had undergone rapid lost.

### Analysis of *PbrPUB* gene family conserved motifs and gene structures

**Fig. 2:**
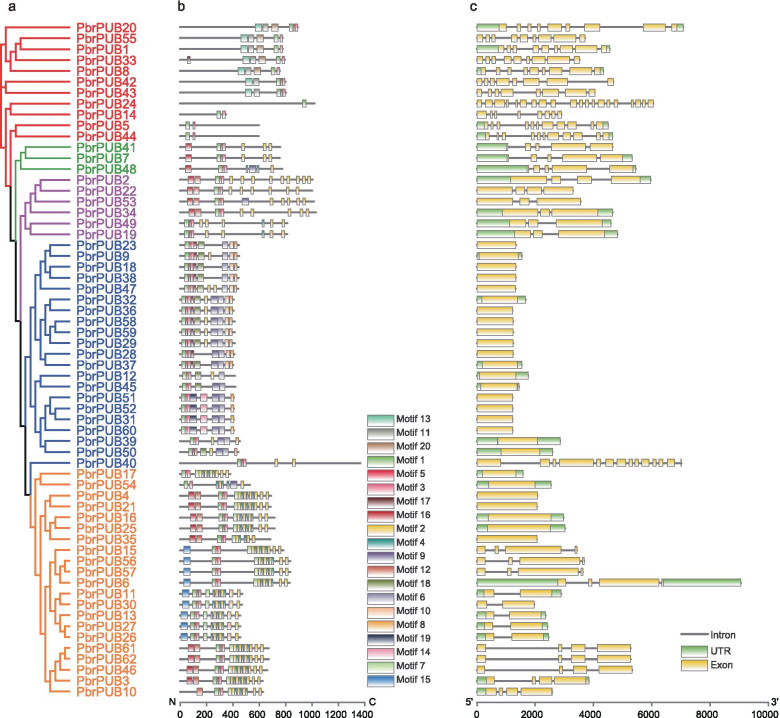
The conserved motifs and gene structure analysis of *PUB* gene family in pear. **a** A Neighbor–Joining (NJ) phylogenetic tree of 62 pear PUB genes. The phylogenetic tree was constructed by Mega-X with 1000 bootstrap. The red branches indicated group I; the blue branches indicated group II; the orange branches indicated group III; the green branches indicated group IV; the purple branches indicated group V; **b** The conserved motifs analysis of *PbrPUB* genes in pear. A total of 20 motifs were predicated by MEME tool, named Motif 1–20. The scale bar indicates 200 aa; **c** The gene structure analysis of *PUB* genes in pear, including UTR, intron and exon. The green rectangles represented UTR; the yellow rectangles represented Exon; the grey lines presented Intron. The scale bar indicates 2 kb

To further verify the classification results of phylogenetic tree, we investigated the conserved motifs and gene structures of *PbrPUB* genes in pear. Totally, twenty motifs were estimated using MEME (Multiple Em for Motif Elicitation) software, and we named as motif 1–20 (Fig. 2a, b, Additional file 1: Figure S1). Among them, motif 1, 3 and 5 were found in all groups, indicating that were highly conserved in all PbrPUB proteins. Based on the SMATR website, we determined that the U-box was comprised of Motif 1, Motif 3 and Motif 5 (Additional file 2: Figure S2). This result provided evidence to support the accuracy of *PUB* genes set identified in our study. Based on the SMATR website, we also found the other conserved domain: ARM and Pkinase domin. The ARM is comprised of motif 2, 4 and 7; the Pkinase is comprised of motif 11, 13 and 20. Generally, most *PbrPUB* members in the same groups had similar conserved motifs. For example, most of the members in Group II contained motif 6, 10 and 8. This result indicated that these three motifs might be key functional domain of Group II PbrPUB proteins, suggesting that these proteins might have conservative functions.

To explore the gene structures of *PbrPUB* genes in pear, we extracted the exon–intron information of 62 *PbrPUB* genes from pear database using in-house scripts. Based on the information, TBtools software was preformed to show the gene structures of *PbrPUB* (Fig. 2c). The number of exon in *PbrPUB* genes was greatly divergent, ranging from 1 to 20. Among 62 *PUB* genes in pear, *PbrPUB24* contained the greatest number of exons (20), while 16 *PUB* genes (25.81%) only contained one exon. Furthermore, the lengths of the exon and intron were differential. There are 30 *PUB* genes have been found to contain untranslated regions (UTR). Similarly, to the result of motif analysis, the *PUB* genes with similar gene structures were cluster into same subclade. For example, most members of Group II only housed one exon. This result indicated that the members of same groups exhibited similar gene structures and conserved motifs. These results from conserved motifs and gene structure analysis provided strong evidence to support the accuracy of the classification result of phylogenetic tree.

### Chromosomal localization and homologous gene analysis of *PbrPUB* genes

**Fig. 3:**
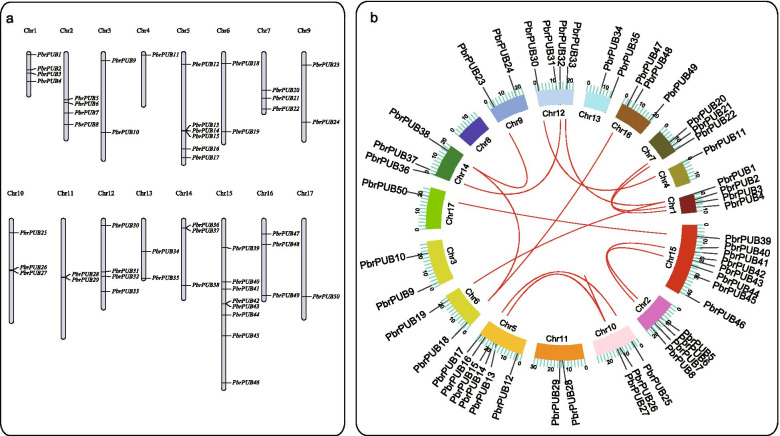
The location distribution and synteny analysis of *PUB* genes in pear genome. **a** The distribution pattern of *PbrPUBs* in 17 pear chromosomes. Due to no *PUB* genes were mapped on Chromosomes 8, we didn’t show it in the Fig. 3; **b** The distribution pattern synteny analysis of *PUB* genes family. The red lines indicated the synteny gene pairs of *PUB* gene family

The chromosome distribution pattern of *PbrPUBs* on genome was predicted by TBtools (Fig. 3a). The location information of *PUB* genes in pear were extracted by our in-house scripts. As a result, a total of 50 *PbrPUB* genes (82.26%) were unevenly mapped on the 17 pear chromosomes, and no member of *PbrPUB* gene family was mapped on chromosome 8. Therefore, we didn’t show chromosome 8 in our Fig. 3. In addition, 12 genes were located on scaffold contigs, and we also didn’t show them in our Fig. 3. Chromosome 15 had the most *PbrPUB* genes, with eight *PbrPUB* genes, followed by chromosome 5 with 6 genes. Chromosome 1, 2, 12 each contained 4 *PbrPUB* genes. Two or three *PbrPUB* genes were mapped on chromosomes 3, 6, 7, 9, 10, 11, 13, 14, and 16. Chromosome 4 and 17 contained only one gene. We also identified the homologous genes of *PUB* gene family using MCScanX software. As result indicate that 16 homologous gene pairs were identified in pear *PUB* gene family, which contained 26 homologous genes. Three homologous gene pairs were detected between chromosomes 5 and chromosomes 10 (Fig. 3b).

### *Cis*-acting elements predication of *PbrPUB* genes

**Fig. 4:**
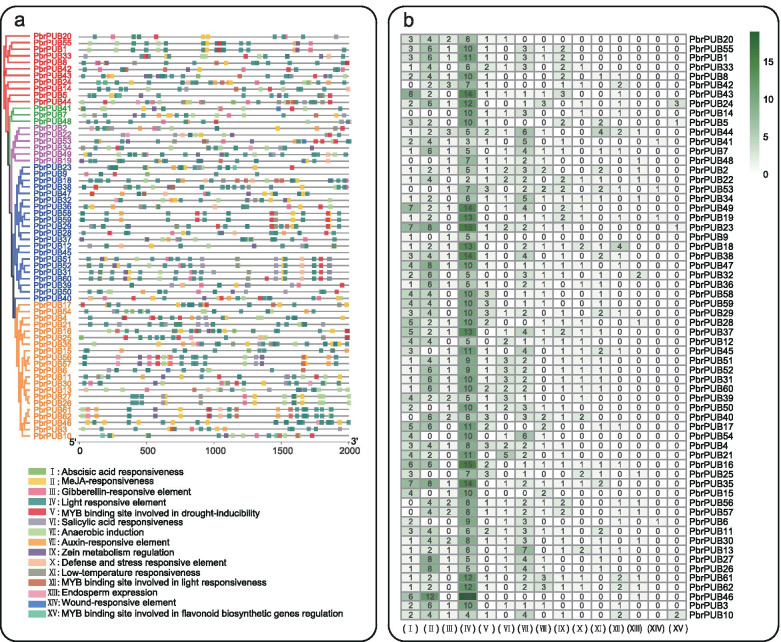
The *cis*-acting elements analysis of putative promoter of 62 *PbrPUB* genes. **a** The distribution pattern of 15 *cis*-acting elements of putative promoter of *PUB* gene family in pear. The phylogenetic tree is same with the phylogenetic tree in Fig. 2a. **b** The number of 15 *cis*-acting elements of putative promoter of *PbrPUB* genes. The color scale at the top right indicated the number of *cis*-acting elements. Green color indicated the number of *cis*-acting elements on *PUB* member. 15 *cis*-acting elements including: (I) Abscisic acid responsiveness; (II) MeJA-responsiveness; (III) Gibberellin-responsive element; (IV) Light responsive element; (V) MYB binding site involved in drought-inducibility; (VI) Salicylic acid responsiveness; (VII) Anaerobic induction; (VIII) Auxin-responsive element; (IX) Zein metabolism regulation; (X) Defense and stress responsive element; (XI) Low-temperature responsiveness; (XII) MYB binding site involved in light responsiveness; (XIII) Endosperm expression; (XIV) Wound-responsive element; (XV) MYB binding site involved in flavonoid biosynthetic genes regulation

*Cis-*acting elements were important clues for the prediction of gene functions. Transcription factors cloud effect the expression level of target genes by binding to the cis-acting element of terget genes in specific biological processes [12]. To further investigate the function of *PbrPUB* genes, we predicted the *cis*-acting element of the putative promoter region of *PbrPUB* genes using PlantCARE databse. As a result, a total of 62 *cis*-acting elements were identified (Additional file 5: Table S2.), and we selected 15 interesting *cis*-acting elements for further analysis. These 15 *cis*-acting were associated with stress, hormone, plant growth and development. As shown in Fig. 4a, some diverse distribution patterns of *cis*-acting elements were observed in the promoter region of *PbrPUB* genes, indicating that the *PUB* gene family of pear particular in various different biology process. Meanwhile, we found that all *PbrPUB* genes contained the *cis*-acting related to hormone regulation, such as salicylic acid, gibberellin (GA), auxin and methyl jasmonate (MeJA) responsiveness elements. Previous study had reported that *DSG1*, which encodes a U-box domain, could regulate cell division and elongation by responding to multiple hormones, such as auxin, salicylic acid and ethylene [41]. ABA responsive element, named as ABRE, is one of most important *cis*-acting element in the promoter sequence of ABA-inducible genes response to ABA treatment [42]. In our study, 55 *PbrPUB* genes were identified as the responsiveness elements of ABA, suggesting that *PUB* gene family might particular in resistance under ABA treatment (Fig. 4b). And in *Arabidopsis*, *AtPUB9*, *AtPUB18*, *AtPUB19*, and *AtPUB44* were identified to involve in ABA response [12]. It is notable that the element related to MYB binding site involved in drought was predicted in 44 *PbrPUB* genes, suggesting that these 44 *PbrPUB* genes might mediated by *MYB* genes response to drought stress. Moreover, there were 30 *PbrPUB* genes have *cis*-acting elements related to cold, suggesting that these 30 *PbrPUB* genes might particular in resistance under low temperature treatment. As we all know, flavonoid biosynthesis is one of the most important phenomenon during the process of response to stress in plant. In this study, we found *PbrPUB10*, *PbrPUB24* and *PbrPUB5* contained MYB binding site involved in flavonoid biosynthetic.

### Tissues-specific expression analysis of *PbrPUB* genes

**Fig. 5:**
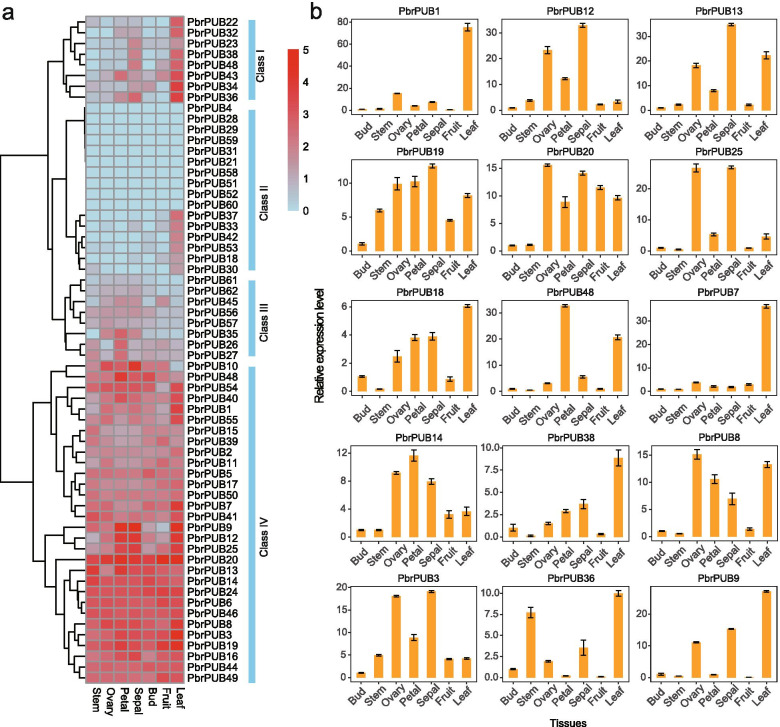
The expression pattern analysis of *PbrPUB* gene family in seven different tissues. **a** The heatmap of expression level of *PbrPUBs* in seven different tissues, including stem, ovary, petal, sepal, bud, fruit and leaf. Pheatmap, an R package, were used to generate the heatmap. The color scale represented the RPKM values normalized by log_2_(RPKM + 1). Red color represented high expression, while blue represented low expression; **b** The expression levels of 15 randomly selected *PbrPUBs* in seven different tissues. Seven tissues are comprised of bud, stem, ovary, petal, sepal, fruit and leaf. The x-axes represented seven different tissues; the y-axes represented the relatively expression of *PUB* genes

To further explore the tissues-specific expressions of *PbrPUB* genes, we collected RNA-seq data of seven tissues of ‘Dangshansuli’ pear from previous study [43]. We used RPKM (reads per kilobase per million) values to estimate the expression level of *PbrPUB* genes. Then, we investigated the expression level of 62 *PbrPUB* genes. Pheatmap, an R package, was used to show the expression patterns of 62 *PbrPUB* genes (Fig. 5a). Based on the expression patterns of 62 *PUB* genes, they were clustered into four main classes. Genes in Class IV exhibited highly expression level in all of seven examined tissues, while genes in Class II exhibited almost no expression in all of seven tissues. Class I was specifically expressed in pear leaf, and a diversity of expression pattern were detected in Class III (Fig. 5a). Among the 62 *PbrPUB* genes, 52 genes (83.87%) were at least expressed in one tissue, even though the transcript abundance of several genes was relatively lower for certain tissues. Approximately 10 non-expressed *PUB* genes (RPKM value less than 1) were identified in all of seven tissues, and they may lost the function during the evolution process of *PUB* gene family in pear. 29 *PbrPUB* genes were expressed in all seven different tissues, indicating that they have various roles in the development of different tissues. Interesting, we found 28 *PUB* genes exhibited highest expression in leaf, suggesting that these 28 genes might involve the development of leaf. Due to leaf is an important plant organ involved resistance, we referred that these 28 *PUB* genes might particular in resistance in the process of pear growth and development.

To verify the transcriptome sequences analysis was reliable, 15 *PbrPUB* genes were randomly selected to conduct a quantitative real-time PCR (qRT-PCR) experiment to investigate the expression levels in seven different tissues of the ‘Dangshansuli’ pear (Fig. 5b). We found that all of 15 *PbrPUB* genes exhibited a diversity of expression patterns in the seven different tissues, suggesting that *PbrPUB* gene family may function in different tissues and participate in diverse metabolic processes. Seven genes (*PbrPUB1*, *PbrPUB3*, *PbrPUB7*, *PbrPUB9*, *PbrPUB18*, *PbrPUB36* and *PbrPUB38*) exhibited a similar expression pattern with a high expression level in leaf tissues, suggesting that *PbrPUB* genes play critical functions during leaf development. And all of these seven genes exhibited highly expression level in leaf in transcriptome data. These results provided further evidence to support our transcriptome sequences analysis was reliable. Interestingly, most of 15 *PUB* genes were highly expressed in reproductive organs, suggesting that *PbrPUB* genes might associate with the development of reproductive organs.

### The expression pattern of *PbrPUB* genes under abiotic stresses

Previous study had extensively reported *PbrPUB* gene family involved in various abiotic stresses [44]. To explore the functions of *PUB* gene family in pear, we detected the expression level of *PbrPUB* in seedling samples (*Pyrus betulaefolia*) subjected to four different stress treatments, including dehydration, low temperature, ABA and salt. 11 *PbrPUB* genes were randomly selected to conduct qRT-PCR experiment. 11 genes are comprised of 2 from group I (*PbrPUB1* and *PbrPUB14*), 4 from group II (*PbrPUB12*, *PbrPUB18*, *PbrPUB36* and *PbrPUB38*), 2 from group III (*PbrPUB3* and *PbrPUB25*), 2 from group IV (*PbrPUB7* and *PbrPUB48*) and 1 from group V (*PbrPUB34*).

**Fig. 6:**
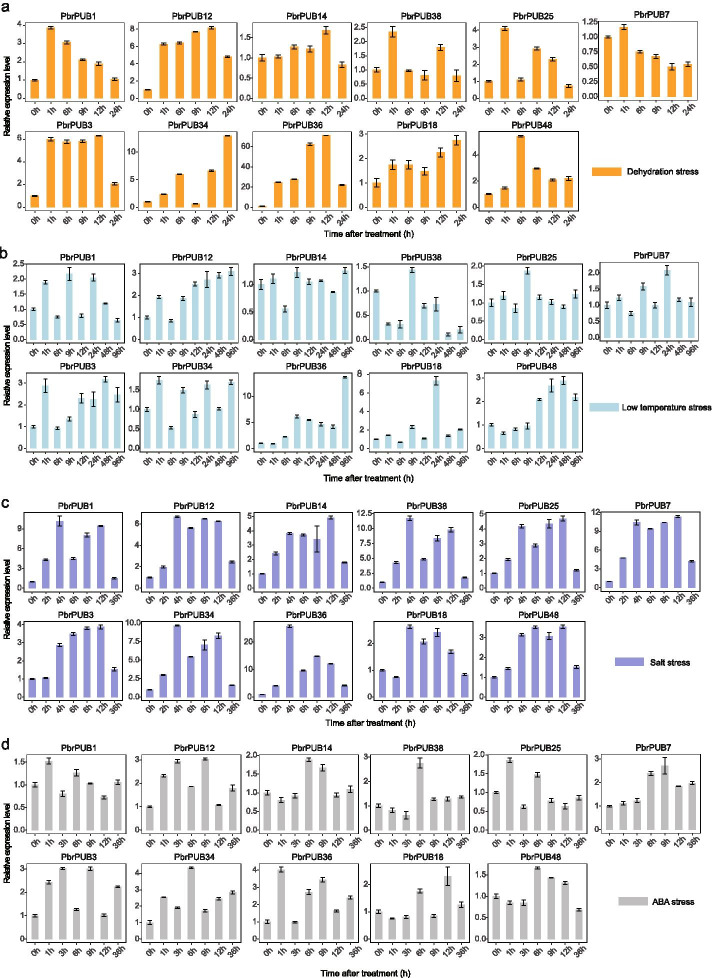
The expression level of 11 randomly selected *PbrPUB* genes in four abiotic stresses. **a** For dehydration treatment, the shoots were placed on dry filter papers for 0, 1, 6, 9, 12 and 24 h; **b** For cold stress, the seedlings were placed in the chamber set at 4 °C for 0, 1, 6, 9, 12, 24, 48 and 96 h; **c** For salt stress, the seedlings were placed in solution containing 200 mM NaCl solution for 0, 2, 4, 6, 8, 12 and 36 h; **d** For ABA stress, The seedlings were dipped in solution containing 100 μM ABA for 0, 1, 3, 6, 9, 12 and 36 h. The x-axes represented time after treatment; the y-axes represented the relatively expression of *PbrPUB* genes

Among the eleven *PUB* genes, 9 *PUB* genes were up-regulated expressed and one *PUB* gene (*PbrPUB7*) was down-regulated expressed under dehydration stress (Fig. 6a). However, *PbrPUB14* was not significantly differential expressed under dehydration stress. Among the 9 up-regulated genes, *PbrPUB18* exhibited highly increased expression level during the process of dehydration treatment, while *PbrPUB12*, *PbrPUB3* and *PbrPUB36* were up-regulated expressed during 12 h dehydration treatment and recovered to normal levels at 24 h. *PbrPUB1*, *PbrPUB38* and *PbrPUB25*, exhibited highest expression level at 1 h, where *PbrPUB12*, *PbrPUB14*, *PbrPUB3* and *PbrPUB36* exhibited highest expression level at 12 h under dehydration treatment. These results suggested that *PbrPUB1*, *PbrPUB38* and *PbrPUB25* respond to dehydration treatment faster than that of *PbrPUB12*, *PbrPUB14*, *PbrPUB3* and *PbrPUB36*. Therefore, *PUB* gene family in pear play vital role in the process of dehydration stress response.

In low temperature treatment (Fig. 6b), we found that 4 genes (*PbrPUB12*, *PbrPUB3, PbrPUB36* and *PbrPUB48*) were up-regulated expressed under cold stress, suggesting that those *PbrPUB* genes might respond to low temperature. *PbrPUB12*, *PbrPUB48* and *PbrPUB36* were highly increased during the 48 h low temperature exposure. The expression level of *PbrPUB3* was reached to double peak at 1 h and 48 h.

In the salt treatment (Fig. 6c), we found that all of the selected 11 *PbrPUB* genes were significantly up-regulated expressed under the 200 mM salt stress treatment. The expression level of *PbrPUB14*, *PbrPUB25*, *PbrPUB3*, *PbrPUB48* and *PbrPUB7* were highly increased during the 12 h salt exposure. Moreover, *PbrPUB1*, *PbrPUB12*, *PbrPUB18*, *PbrPUB34*, *PbrPUB36* and *PbrPUB38* were highest expressed at 4 h under salt stress, suggesting that these 6 *PbrPUB* genes respond to salt treatment actively. We focus on the expression level of *PbrPUB18*. During the 4–8 h, the expression level of *PbrPUB18* was significantly increased, and then it was down-regulated at 12 h, finally recovered normal level at 36 h.

Previous study had reported that *PUB* gene involved in ABA-mediated drought stress responses. In ABA treatment (Fig. 6d), all of 11 *PUB* genes were respond to the ABA stress, and these genes were unregulated expressed at first, then were down-regulated at 36 h after ABA treatment. These results indicated that *PUB* genes play important roles in ABA-regulated pathway. The expression levels of three genes (*PbrPUB1*, *PbrPUB25*, *and PbrPUB36*) were reached to peak at 1 h, suggesting that these three genes were actively responded to ABA stress. Interestingly, we found that *PbrPUB18* was expressed in 6 h and 12 h after ABA treatment.

### Subcellular localization of PbrPUB18 protein

**Fig. 7:**
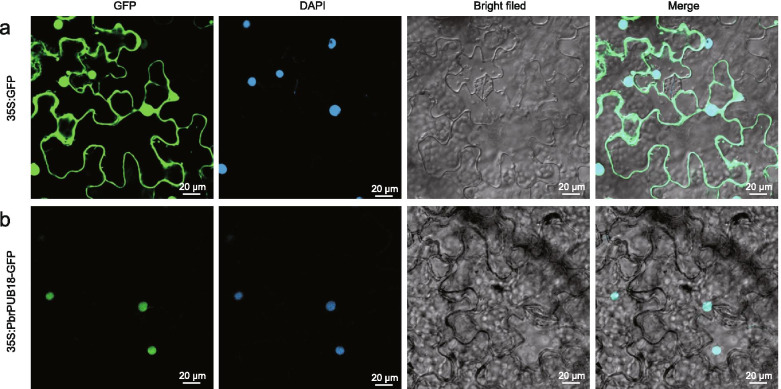
The subcellular localization of PbrPUB18. **a** Tobacco leaf epidermal cells were transiently transformed with constructs containing 35S:GFP vector alone as control; **b** Transient expression of fusion plasmid (35S: *PbrPUB18*-GFP) in tobacco leaf epidermal cells. The nucleus was identified by DAPI staining. Green fluorescence images, DAPI staining mages, blight field images and the merged images are shown from left to right. Scale bars = 20 μm

To further verify the biologic function of *PbrPUB* genes in pear under drought stress, *PbrPUB18* was selected to perform subcellular localization experiment. The green fluorescence of GFP control was found in the membrane and the nucleus (Fig. 7a). In contrast, 35S: *PbrPUB18*-GFP protein was only existed in the nucleus and integrated perfectly with DAPI (4′, 6-diamidino-2-phenylindole) regime (Fig. 7b), suggesting that PbrPUB18 protein was located in the nucleus, which was consistent with our prediction in Table 1.

### Assessment of drought tolerance in transgenic lines of *PbrPUB18*

**Fig. 8:**
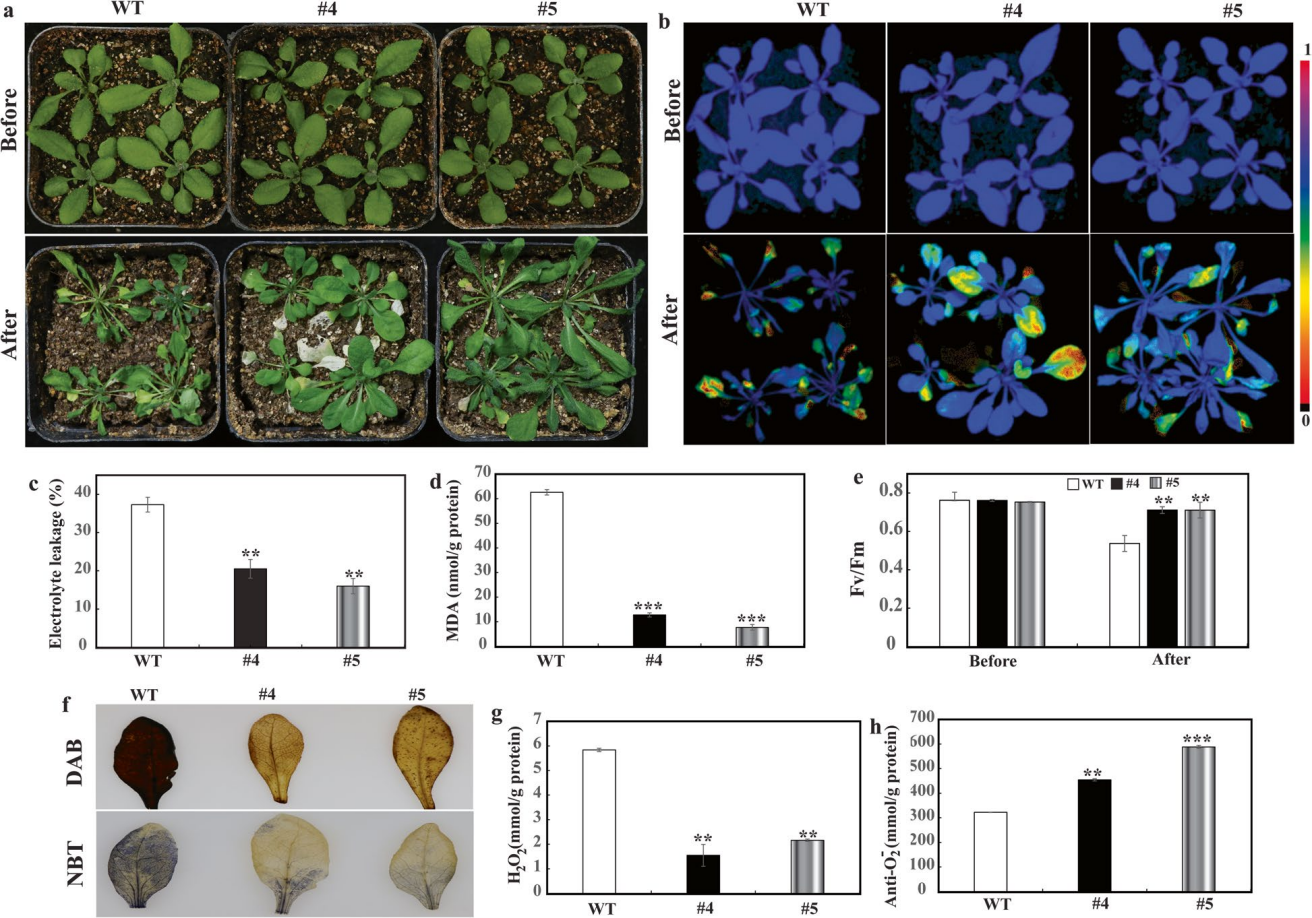
Drought tolerance assay of transgenic *Arabidopsis* plants overexpressing *PbrPUB18*. **a** Phenotypes of 20-day-old transgenic plants and WT before and after 12 days drought stress; **b** Images of (Fv/Fm). The false color code depicted on the right of the image ranges from 0 (black) to 1.0 (purple); Electrolyte leakage (**c**), MDA contents (**d**) in the WT, OE-4 and OE-5 after drought treatment; **e** Fv/Fm of WT, OE-4 and OE-5 before and after drought stress; **f** Histochemical staining with DAB and NBT for detection of H_2_O_2_ and O_2_^−^, respectively, in WT, OE-4 and OE-5 after drought stress for 12 days; Levels of H_2_O_2_ (**g**) and anti-O_2_^−^ (**h**) in *Arabidopsis* WT, OE-4 and OE-5 after drought treatment. Asterisks indicate that the value is significantly different from that of the WT at the same time point (**P < 0.01; ***P < 0.001)

To further confirm the biologic function of *PbrPUB18* gene under drought stress, *Arabidopsis* Col-0 plants (WT) were transformed by the floral dip method [45]. Two overexpression lines OE-4 and OE-5 of *PbrPUB18* were screened out by PCR identification and semi-quantitative PCR at mRNA level. QRT-PCR also verified the expression of *PbrPUB18* in OE-4 and OE-5 far above in WT (Additional file 3: Figure S3). To assess the function of overexpression *PbrPUB18* in *Arabidopsis* on drought tolerance, 20-day-old WT and transgenic lines were conducted to same drought environment (12 days without water). There was no morphological difference between WT and the transgenic lines in the normal condition. After 12 days without water, the two transgenic lines showed more tolerance to the drought stress, as manifested by lesser leaf-wilting symptoms compared with the WT plants (Fig. 8a). In addition, chlorophyll fluorescence measurements were recorded to further verify drought tolerance of WT and the transgenic lines (Fig. 8b). The maximum quantum efficiency of the photochemistry (Fv/Fm) values was not affected by species and growth conditions, but under stress conditions, this parameter decreased significantly. After 12 days drought treatment, the Fv/Fm values of WT was significantly lower than of the two transgenic lines, suggesting WT showed more sensitivity to the drought stress (Fig. 8e). Electrolyte leakage (EL) is extensively used to estimate the cell injury level of plant after drought stress. The EL of two transgenic lines were only approximate 15%–20% compared to WT (37.3%), suggesting that WT suffered more severe membrane damage than transgenic lines of *Arabidopsis* by overexpressing *PbrPUB18* (Fig. 8c). The transgenic plants displayed significantly lower malondialdehyde (MDA) contents than WT exposure to drought condition (Fig. 8d). These results indicated that two transgenic lines of *PbrPUB18* suffered to relatively lighter extent oxidative stress.

Histochemical staining shown that the leaves of WT exhibited more deeper staining compared with that of OE-4 and OE-5 after drought stress (Fig. 8f), suggesting that WT type accumulated more ROS (H_2_O_2_ and O_2_^−^). Similar to staining results, quantitative measurements further demonstrated that two transgenic lines exhibited significant lower H_2_O_2_ contents than that of WT type (Fig. 8g). Moreover, anti O_2_^−^ contents in the two transgenic lines were significantly higher than those of WT (Fig. 8h). These results indicated that *PbrPUB18* could enhance drought resistance.

## Discussion

### Genome-wide and phylogenetic analysis of *PbrPUB* genes in pear

As a family of ubiquitin ligases, U-box genes encode a conserved U-box motif of about 70 amino acids and regulated the ubiquitination of the substrates [23]. U-box genes were widely distributed in the plants and reported to participate in many biological processes including plant hormone signaling regulations [6], self-incompatible or pseudo-self-compatibility regulations [18] as well as in biotic stress[19–21] and abiotic stress [5, 22, 23]. Due to *PUB* gene play an important role during plant development, *PUB* genes have been identified in different plant species, such as *Arabidopsis thaliana* (61) [9, 10], rice (77) [11], tomato (62) [12], cotton (93) [13], and banana (91) [14]. Pear, one of Rosaceae fruit trees, is widely cultivated all over the world. However, the analysis related to *PUB* genes in pear was poor until now. In the present study, 62 genes were identified as *PUB* gene family in pear using bioinformatics analysis, and the number of *PUB* gene in pear is similar to that of *Arabidopsis thaliana* (61), tomato (62) and apple (69).

Phylogenetic tree analysis indicated that a total of 185 PUB protein members in these three species (containing 62 pear, 62 tomato, and 61 *Arabidopsis*) were categorized into five groups (I-V). The results of phylogenetic tree were similar with other species [12, 14]. For example, 125 *GmPUB* genes in soybean proteins were classified into six groups using phylogenetic tree analysis [17]. In apple, 69 *U-box* genes were clustered into seven groups [18]. Through the phylogenetic relationship analysis, it showed that *PbrPUB*s exhibited closer relations with *SIU-boxs* compared with *AtPUBs*. This result was consistent with the fact that pear and tomato exhibited closely relationship than *Arabidopsis*. Although the number of *PUB* genes was similar in three species, we found that the *Arabidopsis* genes of Group I of had undergone rapid expansion and of Group II had undergone rapid lost. In addition to the U-box domain, 62 PbrPUB proteins are found to bind to different domains including armadillo (ARM) repeats, the tetratricopeptide (TPR) domain and WD40 repeats. The majority of PUB proteins that have been elucidated for biological functions are from the U-box proteins with ARM repeats [18]. The ARM repeats have been shown primarily mediating the interaction with substrates, suggesting that interaction make the substrates available for ubiquitination [23]. 25 member of *PUB* genes in pear only housed U-box domain, and 25 members housed both U-box and ARM domain. Moreover, TPR domain was found in *PbrPUB14* gene and WD40 repeats was found in *PbrPUB40* gene.

### The function predication of *PUB* gene family in pear based on *cis*-acting and specific-tissues expression analysis

The *cis*-acting analysis of putative promoter indicated that the *PUB* gene family was involved in stress-related mechanisms, hormonal regulation, growth and development. Previous studies had reported that *PUB* were responded with ABA. For instance, *AtPUB44* could regulate the biosynthesis of ABA through ubiquitinating the AAO3 (abscisic aldehyde oxidase 3) via 26 proteasomes [35]. In additional, one transcription factor of ABI3 was regulated by *AtPUB9* and increased the ABA sensitivity of *Arabidopsis* during seedling germination [46]. *AtPUB18*, *AtPUB19* and *AtPUB44* were found to directly interrupt the biosynthesis of ABA. In our study, 55 genes contained the ABA responsive element on the putative promoter region. Especially, we found that eight ABA responsive elements were identified in the promoter region of *PbrPUB*43. This result indicated that *PUB* gene might play significant role during ABA signal transduction in pear. In *Arabidopsis* and *Nicotiana*, the expression of *PUB* genes was regulated by abiotic and biotic stress [18]. Previous study had reported that MYB transcription factor could regulated the expression level of resistance genes. For example, *PbrMYB21* can specifically bind to the MYB recognition sites in promoters of *PbrADC* and played a positive role in drought tolerance [47]. In here, we also found MYB binding site involved in drought induction responsive element was identified in the promoter region of *PUB* genes in pear, suggesting that *PUB* genes might be regulated by related transcription factors mediating the drought stress signaling. We also found abscisic acid responsive element, defense and stress responsive element, low temperature responsive element, wound responsive element in the promoter region of *PUB* genes in pear. These results indicated that *PUB* genes might involve in a diverse of biology process during pear growth and development.

Based on RNA-Seq data and qRT-PCR experiment, we investigate the expression level of *PbrPUBs* in seven tissues. Among the 62 members of *PUB* gene family in pear, 29 *PbrPUB* genes were expressed in all seven different tissues. Additionally, 72.58% of *PbrPUB*s were expressed in pear sepal. Whereas of *PbrPUB*s expressed in all tissues, 45.16% were highest in leaves, suggesting these genes may function in the development of pear leaves. qRT-PCR analysis shown that 15 *PbrPUB*s have highly expression level in ovary, leaf, sepal and petal, suggesting that *PbrPUB* genes may function in the development of these four tissues.

### Roles of *PbrPUB* genes in response to different abiotic stresses

Previous studies have reported that *PUB* genes involved in the process of stress responses [32, 33, 48, 49]. A large number of *PUB* genes were induced expressed during abiotic stress conditions [19]. In this study, the differential expression levels of 11 *PbrPUB* genes under various abiotic stresses were investigated by using qRT-PCR, including dehydration, low temperature salt and ABA stress. From the result, *PbrPUB12*, *PbrPUB3*, *PbrPUB36* and *PbrPUB48* were significantly up-regulated expressed under four treatment, suggesting these three genes could response to dehydration, ABA, cold and salt stress. *PbrPUB7* was down-regulated expressed under dehydration stress, suggesting that *PbrPUB7* might negatively regulate the response process of dehydration.

In *Arabidopsis*, the function of most *PUB* members from Group II were widely investigated in abiotic stresses process of plant. *AtPUB22/AtPUB23* are negative regulators mediating drought responses in the ABA-independent pathway [34]. *AtPUB25* and *AtPUB26* participated in plant response to low temperature signal by regulating the protein stability of *MYB15*, a negative transcription factor in CBF signaling pathway [32]. *AtPUB30* negatively regulates salt tolerance by facilitating BRI1 KINASE INHIBITOR 1 (BKI1) degradation [50]. In addition, *MdPUB29*, highly homologous with *AtPUB29*, may positively regulate salt tolerance [38]. We inferred *PbrPUBs* in Group II may also related to abiotic stress. Drought is one of most critical stresses and could significantly affect the growth of plant. And in our study, we found the expression level of *PbrPUB* genes from Group II (*PbrPUB12*, *PbrPUB18*, *PbrPUB36* and *PbrPUB38*) were significant up-regulated after dehydration treatment. In order to verify the role of *PbrPUBs* in drought stress, *PbrPUB18* was selected for further functional identification.

Subcellular localization experiment suggested that PbrPUB18 protein was located on the cell nucleus. This result indicated that *PbrPUB18* might act biology function at the cell nucleus. Previous study indicated that MYB15 act as a negative regulator factor during freezing stress, and *PUB25* and *PUB26* can improve the resistance in cold stress by accelerate the degradation of *MYB15* [32]. These results suggesting that *PbrPUB18* might degradate transcription factors to positively regulate the plant resistance, for example, MYB transcription factors. Heterologous over-expression of *PbrPUB18* in *Arabidopsis* shown better physiological traits, such as lower MDA content, lower EL and higher Fv/Fm than WT in 12 days drought treatment suggesting that overexpression of *PbrPUB18* could enhance drought resistance. ROS content analysis indicated that lower levels of H_2_O_2_ and higher levels of anti O_2_^−^ in the transgenic lines, suggesting that the tolerance may be ascribed to more robust activation of ROS scavenging system. *AtPUB19* and *AtPUB18* act negative roles on ABA signaling pathway downstream of H_2_O_2_ [34]. But the cellular mechanism by what *PbrPUB18* regulating drought responses remained unclear, and needed to be explored in the future study. Summary, we systematically identified the *PUB* gene family in pear, and further function identification laid a foundation for the functional study of *PUB* genes of pear in future.

## Conclusions

In our study, a total of 62 *PbrPUB* members were identified in Chinese white pear (*Pyrus bretschneideri*) genome, and were unevenly distributed on 17 pear chromosomes. According to phylogenetic tree analysis, *PbrPUBs* were divided into five groups. The conserved motifs and gene structures analysis provided strong evidence to support the result of classification. *Cis*-acting element analysis indicated that *PUB* genes might participate in diverse biological processes, especially in response to abiotic stresses and phytohormones. Transcription sequencing data from different seven tissues exhibited diverse of expression level of *PbrPUB* genes. Further qRT-PCR was used to identify candidate genes associated with abiotic stresses. In addition, *PbrPUB18* was cloned and functionally identified. Subcellular localization revealed PbrPUB18 protein was located on cell nucleus. Heterologous over-expression of *PbrPUB18* in *Arabidopsis* indicated that the over-expression of *PbrPUB18* could enhance resistance in drought treatment. But the cellular mechanism of *PbrPUB18* regulating drought responses was needed to be explored in the future study.

## Materials and methods

### Genome identification of *PUB* gene family members in Chinese white pear

To identify the potential members of the *PUB* gene family, we firstly downloaded the pear genome (*Pyrus bretschneideri*) from NCBI database [40]. Then, the seed file of U-box domain (PF04564) was used to search the candidate *PUB* genes in pear protein database using HMMsearch software. All candidate PbrPUB proteins obtained from the result of HMMsearch were further submitted to SMART website (http://smart.embl-heidelberg.de/) to determine completeness of U-box conserved domain. In addition, the pI and MW of PbrPUBs protein were calculated by ExPASy. Then, we also investigated subcellular localization of PbrPUBs using Cell-PLoc 2.0 [51].

### Phylogenetic analysis of PbrPUB proteins

Firstly, we collected PUB protein sequences of *Arabidopsis*, tomato and pear [10, 12]. A total of 185 PUB protein sequences were download. Second, ClustalW function of MEGA-X software was used to perform sequence alignment using these 185 PUB protein sequences. Third, the phylogenetic tree was constructed by MEGA_X (Method, NJ; Bootstrap, 1000) [52]. Finally, we used Evolview tool (https://evolgenius.info//evolview-v2/#login) to edit the phylogenetic tree of PUB proteins [53].

### Gene structures, motif analysis and *cis-*acting elements analysis

To identify and visualize the structural organization (introns, exons and UTR) of the pear *PUB* gene family, the information of gene structure was extracted from whole genome database of pear using in-house scripts. The novel conserved motifs of *PbrPUB* genes were identified by MEME suite (http://meme-suite.org/tools/meme). A total of 20 motifs and a width limit of 200 amino acids were used for the analysis with other default parameters. TBtools were used to visualize the results of gene structure and conserved motif analysis [54]. The 2000-bp region of upstream of *PbrPUBs* (same strand) were defined as putative promoter sequence. We obtained the promoter sequence of *PbrPUBs* using getfasta function in Bedtools [55]. *Cis*-acting elements of *PbrPUBs* were predicted by PlantCare tools [56]. According to function annotation of *cis*-acting element (Additional file 5: Table S2), the interesting elements were obtained for further study, and the *cis*-acting element with same function annotation were integrated to same group.

### Synteny analysis and chromosomal localization

The homologous gene pairs of *PbrPUBs* were identified by blast software with all-vs-all blast strategy. Then, the synteny regions were identified by MCScanX using the result of all-vs-all blast [57]. We plotted circos picture to show the distribution of synteny gens pairs [58]. The chromosome location analysis was conducted by TBtools [54].

### Gene expression analysis of *PbrPUB* on the RNA-Seq Data

The RNA-seq data of the ‘Dangshansuli’ in seven different tissues were download from NCBI [43]. Fastp software was used to perform quality control and filter. Bowtie2 and Tophat2 software were used to perform reads mapping. The RPKM values were measured by featureCount software and in-house python scripts. Then, we used Heatmap.2 package to show the expression pattern of *PbrPUBs* (log_2_ (RPKM + 1)) [59].

### Plant materials and stress treatments

The seeds of *Pyrus betulaefolia* were collected from our experimental field (the pear germplasm orchard of the Center of Pear Engineering Technology Research situated at Hushu in Nanjing). Then, the seeds of *Pyrus betulaefolia* were cultivated in the National Center of Pear Engineering Technology Research (Nanjing Agricultural University, Nanjing). To further explore the function of *PbrPUBs* during abiotic stresses, the seedlings were subjected to four different abiotic stresses, including dehydration, low temperature, salt and ABA treatment. The method of abiotic treatment was development from our previous method with minor revision [60, 61]. In dehydration treatment, six time points (0, 1, 6, 9, 12 and 24 h) were selected to collected leaves of pear seeding under stress. In cold treatment, eight time points were selected (0, 1, 6, 9, 12, 24, 48 and 96 h). In salt treatment, seven time points were selected (0, 2, 4, 6, 8, 12 and 36 h). In ABA treatment, seven time points were selected (0, 1, 3, 6, 9, 12 and 36 h).

### QRT-PCR analysis

Total RNA of leaves materials under stress was extracted using Plant Total RNA Isolation Kit Plus (FOREGENE, China). Then, PrimeScript™ RT reagent kit (Takara Bio, China) was used to reverse transcribe RNA to cDNA. QRT-PCR analysis was conducted on Roche LightCycler® 480 II (Roche, Mannheim, Germany) using LightCycler® SYBR GREEN I Master Mix kit (Roche, China). We designed fifteen pairs specific primers (Additional file 4: Table S1) using Primer5.0 software and checked by using NCBI online software (https://www.ncbi.nlm.nih.gov/). The reaction system and protocol of qRT-PCR were consistent with our previously study [59, 62]. The relatively expression level of *PbrPUBs* were estimated using 2^−∆∆CT^ method [63]. The pear *Tubulin* gene (No. AB239681) was selected as an internal reference for *Pyrus betulaefolia*, and the *Actin* gene (No. AY063980) was selected as an internal reference for *Arabidopsis*.

### Subcellular localization

The open read frame (ORF) of *PbrPUB18* lacked of stop codon were cloned from the cDNA of *Pyrus betulaefolia* using primer pairs (GSP16, Additional file 4: Table S1). We conducted a 35S: *PbrPUB18*-GFP fusion vector based on previous study [64]. We transformed 35S: *PbrPUB18*-GFP fusion vector into *Agrobacterium tumefaciens* strain GV3101, and we also transformed 35S: GFP as control group[64]. The fluorescence signal was observed with a confocal laser scanning microscope (LSM800, Germany) after 72 h post infiltration and the position of nucleus was revealed by staining with DAPI.

### *Arabidopsis* transformation and characterization of transgenic plants

*Arabidopsis thaliana* ecotype Columbia Col–0 plants were transformed for heterologous over-expression *PbrPUB18* by using the floral dip method [45]. And *Agrobacterium tumefaciens* suspension containing the vector 35S: *PbrPUB18*-GFP (OD_600_ = 0.80) was used for transformation. T0 seeds were identified by Murashige and Skoog (MS) solid mediumwith 20 mg·L^−1^ hygromycin and then verified by PCR analysis using specific primers pair (GSP17, Additional file 4: Table S1). According to previous research [60, 61], semi-quantitative RT-PCR and qRT-PCR was used to further analyze the transcript levels of *PbrPUB18* in T1 plants with primers pair (GSP18 and GSP5, Additional file 4: Table S1). Two overexpressing lines (OE-4 and OE-5) of *PbrPUB18* were choosed to cultivate T3 homozygous plants for the further drought tolerance assay.

### Assessment of drought tolerance in transgenic lines

To verifiy the drought tolerance of transgenic lines of *PbrPUB18*, the seedlings (20 days) of transgenic lines of *PbrPUB18* and WT (control) were exposed to drought treatment (withholding water) for 12 days. Then, we collected the leaves samples from WT and transgenic lines for estimating phonotype data, including EL, MDA, ROS content. Electrolyte leakage was measured by conductivity monitor (TOADKK, Japan) [65]. Following the instructions of manufacturer, we measured the MDA and ROS (H_2_O_2_ and O_2_^−^) content by specific analytical kits (Nanjing Jiancheng Bioengineering Institute, Jiangsu, China), repectively. To further observed ROS (H_2_O_2_ and anti-O_2_^−^) level, we also used DAB and NBT to perform histochemical staining [66]. The IMAGING-PAM chlorophyll fluorometer was concucted to monitor the level of the chlorophyll fluorescence using ImagingWin software (Walz; Effeltrich, Germany). The detail parameters and the estimate method of Fv/Fm values were described by Woo et al. [67].

### Statistical analysis

In our study, each phonotype data of abiotic stress and expression profile of qRT-PCR were repeated at least three times. The data were shown in figures as mean ± standard error (SE). All statistical analyses were performed in R language. *T-test* function in R were used to test the significance levels of phonotype data between treatment and control groups (*P < 0.05, **P < 0.01 and ***P < 0.001).

## Supplementary Information

**Additional file 1: Figure S1.** The logos of 20 conserved motifs predicted in our study.

**Additional file 2: Figure S2.** The distribution of domain of *PUB* genes in pear. The conserved domains were predicted by SMART tools.

**Additional file 3: Figure S3.** Molecular identification of transgenic *Arabidopsis* plants overexpressing *PbrPUB18*. (a) Genomic PCR identification of the plants using specific primers of *PbrPUB18*. M, DNA marker (DL 5000); + , positive control (gene plasmid); WT, untransformed plants. Numbers on the top of the gel panels indicate the transgenic lines; (b) Semi-quantitative RT-PCR analysis of the transcript levels of *PbrPUB18* in six transgenic lines and WT. M, DNA marker (DL 2000); WT, untransformed plants; (c) The expression level of *PbrPUB18* in WT and two transgenic lines. *Actin* was used as an internal control gene for normalizing the expression levels; Asterisks indicate that the value is significantly different from that of the WT at the same time point (* < 0.05; **P < 0.01; ***P < 0.001).

**Additional file 4: Table S1.** Primer sequences used for expression analysis, cloning, subcellular localization, vector construction and transgenic confirmation.

**Additional file 5: Table S2.** The *cis*-acting element analysis of *PbrPUB* gene family in pear.

## Data Availability

The transcriptome sequencing raw data from seven different pear tissues have been deposited at NCBI (https://www.ncbi.nlm.nih.gov/bioproject/?term=PRJNA498777).

## Abbreviations

PUB: Plant U-box gene
UPS: Ubiquitin/26S proteasome system
Ub: Ubiquitin
E1: Ubiquitin-activating enzyme
E2: Ubiquitin-conjugating enzyme
E3: Ubiquitin ligase
HECT: Homology to E6-Associated Carboxy-Terminus
RING: Really Interesting New Gene
CRLs: Cullin–RING ligases
UFD2: Ubiquitin fusion degradation protein-2
LRRR1: LEUCINE‐RICH REPEAT PROTEIN1
KIN7: KINASE7
ABI1: ABA-INSENSITIVE 1
AAO3: Abscisic aldehyde oxidase 3
UTR: Untranslated regions
ABA: Abscisic acid
MeJA: Methyl jasmonate
GAs: Gibberellin
ABRE: ABA responsive element
RPKM: Reads per kilobase per million
qRT-PCR: Quantitative real-time PCR
GFP: Green fluorescent protein
DAPI: 4′, 6-Diamidino-2-phenylindole
Fv/Fm: The maximum quantum efficiency of the photochemistry
WT: Wide type
MDA: Malondialdehyde
EL: Electrolyte leakage
DAB: 3, 3′-Diaminobenzidine
NBT: Nitro-blue tetrazolium chloride
ROS: Reactive oxygen species
TPR: Tetratricopeptide
ARM: Armadillo
