# Supplementary material for: Genome-wide identification and functional analysis of U-box E3 ubiquitin ligases gene family related to drought stress response in Chinese white pear (Pyrus bretschneideri)
Source: BMC Plant Biol. 2021 May 26;21:235. doi: 10.1186/s12870-021-03024-3 (PMC8152096; doi:10.1186/s12870-021-03024-3)
Supplement: Supplementary file 1 — Additional file 1: Figure S1. The logos of 20 conserved motifs predicted in our study. [file 12870_2021_3024_MOESM1_ESM.pdf]

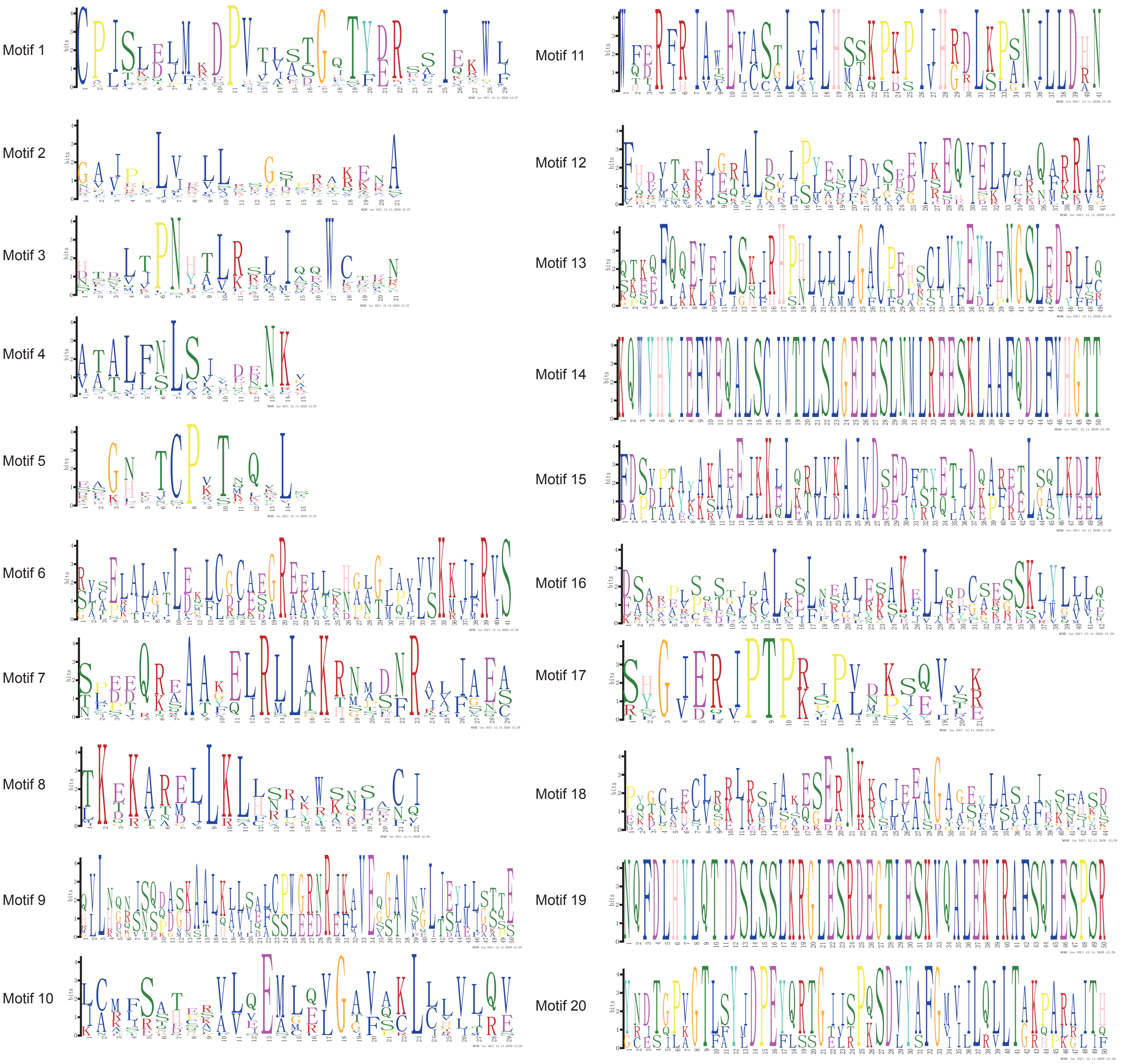

Motif Consensus

Motif 1: CPISLELMKDPVTLSTGQTYDRSSIZKWL
Motif 2: GAIPPLVELLRSGSPRAKEBA
Motif 3: HTDLTPNHTLRSLIQZWCTEN
Motif 4: ATALFNLSIYDENKV
Motif 5: EAGNRTCPVTNQVLS
Motif 6: RVAELALAVLEKLCGCEEGREELLKHGAGIAVLVKKILRVS
Motif 7: SLEEQREAAKELRLLAKRNMDNRALIAEA
Motif 8: TKEKAREJLKLLSIKWKNSACI
Motif 9: QVLNQNISQDASKAAJKLLSELCPWGRBRIKAVEQGAVNVLIELLJGTTE
Motif 10: JCMFSATERVLQEMLZVGAVAKLLLVLQV
Motif 11: WFERFRIAWEVASTLVFLHSSKPKPIIHRDLKPANILLDHN
Motif 12: FHDVTKEJERALDVJPLENLDISEEVKEQVELLQAQARRAE
Motif 13: QTKEFQQEVEILSKIRHPHLLLLLGACPEHSCLVYEYMENGSLEDRLLQ
Motif 14: KQWYHYIEFVEQALSCIVTLLSLGELESLNMLREESKLAAFQDLFVHGTT
Motif 15: FDSVPTALAKAEEJKKELKRLVKAIVDDEDFSTETLDKAFETLSALKDLK
Motif 16: DSAEPLSESAIKALKELNEALEKAKELLQYCSESSKLYLLLQ
Motif 17: SHGIERIPTPKIPVBKSZVVK
Motif 18: PQGCLECLRKJKSIAKESERNKKCIEEAGAGEYLAAIINSFASB
Motif 19: NQFDLHYLQTIDSLSSLKRGLESRDEGTLESKVQALEKIRAFSQLESPSR
Motif 20: YNDTGPVGTLSYIDPEYQRTGIISPQSDVYAFGMVILQLLTAKPARAITH
