# Supplementary figures and images for: Genome-wide identification and functional analysis of U-box E3 ubiquitin ligases gene family related to drought stress response in Chinese white pear (Pyrus bretschneideri)
Source: BMC Plant Biol. 2021 May 26;21:235. doi: 10.1186/s12870-021-03024-3 (PMC8152096; doi:10.1186/s12870-021-03024-3)

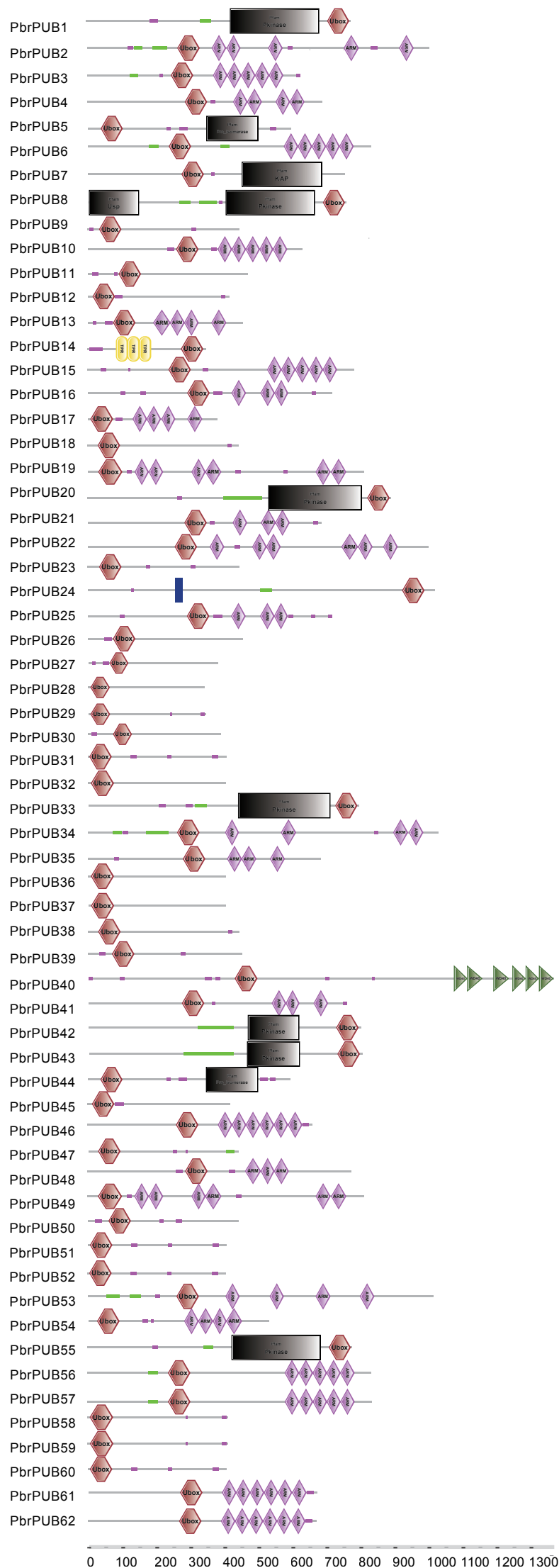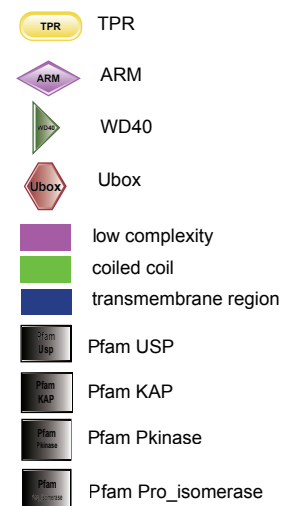

Supplement: Supplementary file 2 — Additional file 2: Figure S2. The distribution of domain of PUB genes in pear. The conserved domains were predicted by SMART tools. [file 12870_2021_3024_MOESM2_ESM.pdf]

**a**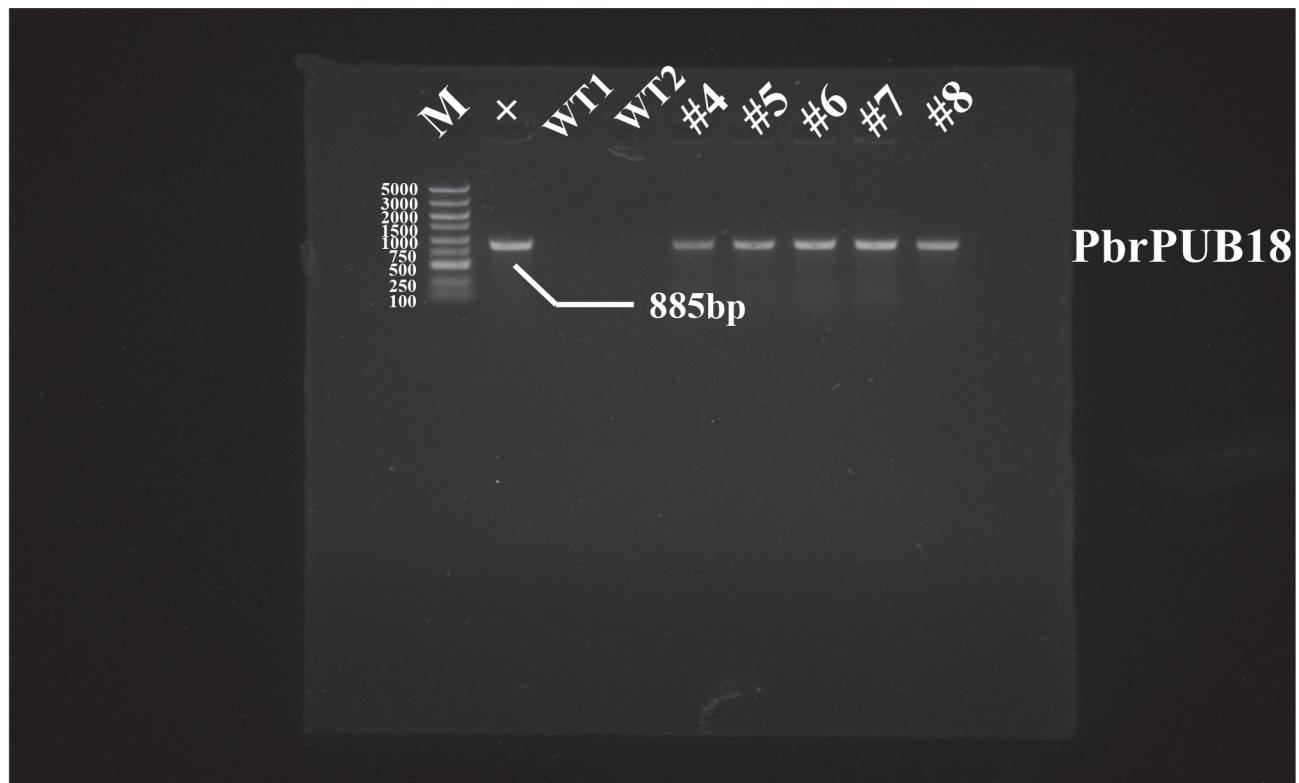**c**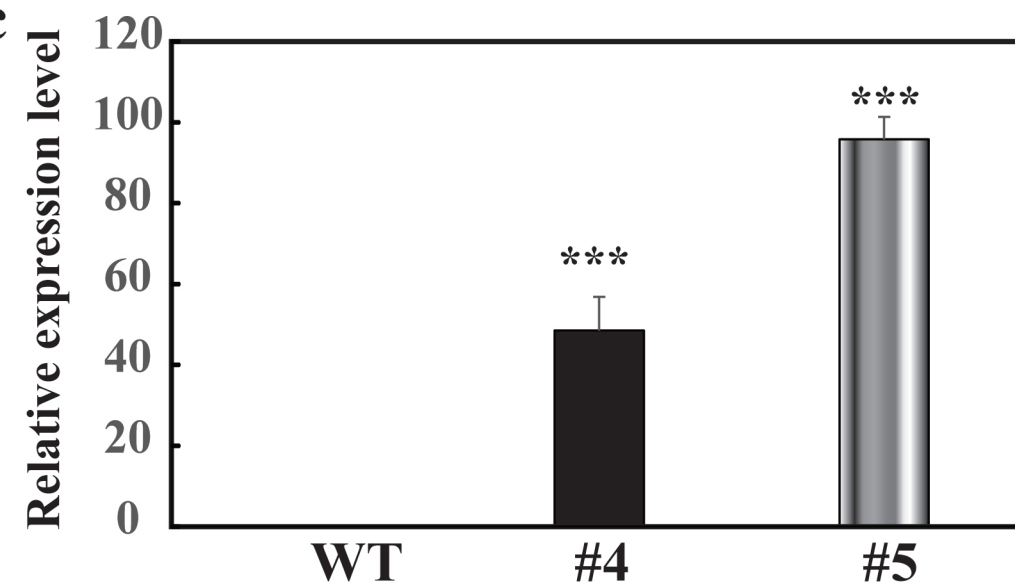**b**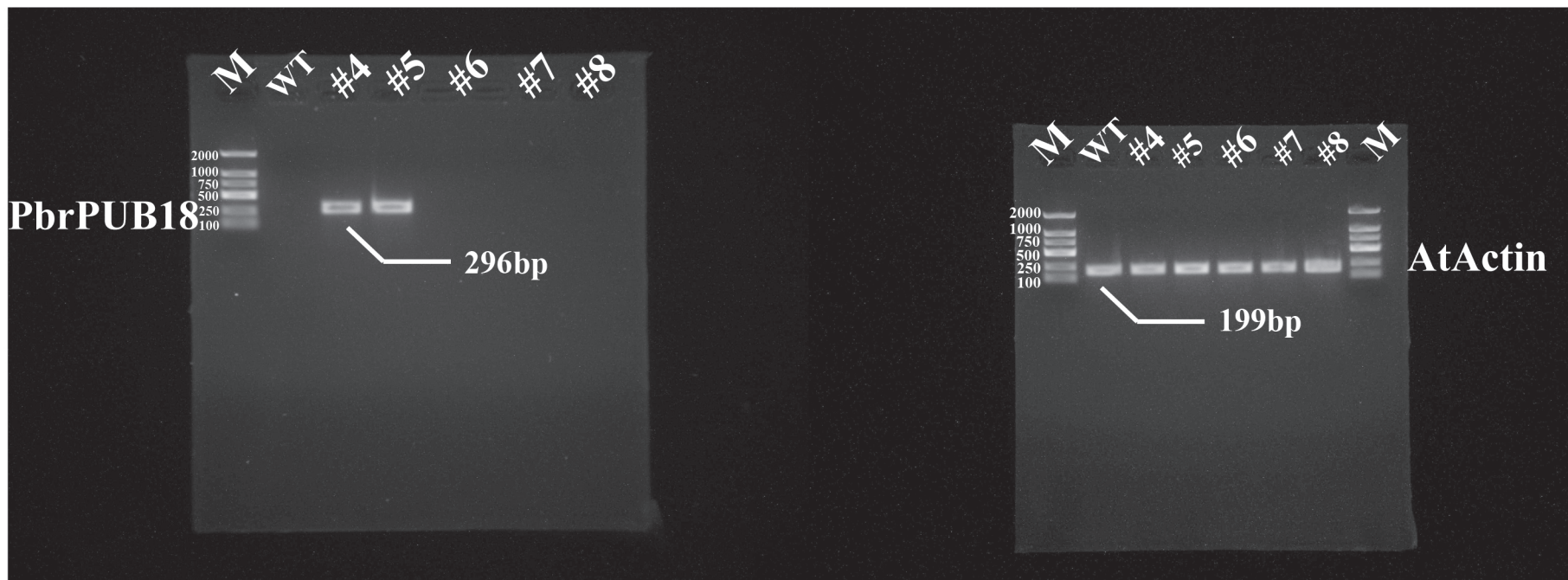

Supplement: Supplementary file 3 — Additional file 3: Figure S3. Molecular identification of transgenic Arabidopsis plants overexpressing PbrPUB18. (a) Genomic PCR identification of the plants using specific primers of PbrPUB18. M, DNA marker (DL 5000); + , positive control (gene plasmid); WT, untransformed plants. Numbers on the top of the gel panels indicate the transgenic lines; (b) Semi-quantitative RT-PCR analysis of the transcript levels of PbrPUB18 in six transgenic lines and WT. M, DNA marker (DL 2000); WT, untransformed plants; (c) The expression level of PbrPUB18 in WT and two transgenic lines. Actin was used as an internal control gene for normalizing the expression levels; Asterisks indicate that the value is significantly different from that of the WT at the same time point (* < 0.05; **P < 0.01; ***P < 0.001). [file 12870_2021_3024_MOESM3_ESM.pdf]
